# Etiologic Types and Complications of Diabetes Mellitus in Newly Diagnosed Patients at Health Institutions in Bulawayo, Zimbabwe: Protocol for a Cross-Sectional and Prospective Observational Study

**DOI:** 10.2196/74186

**Published:** 2026-01-06

**Authors:** Rudo Gwini, Elopy Sibanda, Desmond Mwembe, Fraser James Pirie, Ayesha A Motala

**Affiliations:** 1 Department of Medicine, Faculty of Medicine, National University of Science and Technology Zimbabwe, Vera Road, Mzilikazi, P.O. Box AC939 Ascot, Bulawayo, Zimbabwe, 263 772119623; 2 Department of Statistics and Operations Research, Faculty of Applied Sciences, National University of Science and Technology, Bulawayo, Zimbabwe; 3 Department of Diabetes and Endocrinology, School of Clinical Medicine Nelson R Mandela, University of KwaZulu-Natal, Durban, South Africa

**Keywords:** type 1 diabetes, type 2 diabetes, diabetes complications, etiologic type, autoantibodies, C-peptide

## Abstract

**Background:**

The prevalence of diabetes mellitus is increasing in sub-Saharan Africa. Data on the prevalence of diabetes in Zimbabwe are scarce, and the etiologic types of diabetes are not well characterized. Classification of diabetes in Zimbabwe relies on clinical criteria at the time of diagnosis, and more detailed phenotype data are lacking. Furthermore, the prevalence of complications at diagnosis of diabetes and the incidence of complications during follow-up are not well documented in Zimbabwe.

**Objective:**

The primary aim of this study is to characterize the etiological types of diabetes in adult and adolescent patients with newly diagnosed diabetes in Bulawayo, Zimbabwe. The secondary objectives are to determine the prevalence of chronic complications of diabetes among adult and adolescent patients with newly diagnosed diabetes and to determine the incidence and risk factors for the development of diabetes complications after a 2-year follow-up in patients with type 2 diabetes who are free of complications at baseline.

**Methods:**

This is a cross-sectional and prospective observational study. The cross-sectional (phase 1) study was conducted in patients presenting for the first time to the diabetes service at 2 referral hospitals and 2 diabetes clinics in Bulawayo, Zimbabwe. Data collected from consenting participants included demographic data, social and medical history, and clinical examination. Laboratory tests included serum urea, creatinine and electrolytes, liver function tests, lipids, plasma glucose, glycated hemoglobin, serum C-peptide, spot urine (dipstick, albumin, and creatinine), and β-cell antibodies (antiglutamic acid decarboxylase, anti-islet antigen, anti-insulin antibodies, anti-islet cell antibodies, and antizinc transporter 8 antibodies). All patients had retinal photography, a 12-lead electrocardiograph, and measurement of carotid intima-media thickness and arterial stiffness. Determination of the incidence of diabetes complications will be conducted through a 2-year follow-up (encompassing 6-mo review) of a subgroup of patients with type 2 diabetes and no diabetes complications at the time of enrollment. At each 6-month visit, in addition to all variables collected at baseline, data on diabetes management and drug therapy compliance will be obtained.

**Results:**

Data collection commenced in October 2021, with 323 participants recruited. Data analysis for phase 1 is ongoing. The study will be completed in October 2026. The results will describe the spectrum of diabetes and complications found at diagnosis (phase 1) and the incidence and risk factors associated with the development of complications of diabetes (phase 2).

**Conclusions:**

The study will provide data on etiologic types of diabetes in patients presenting to health facilities in urban centers in Bulawayo, Zimbabwe. In addition, data on diabetes complications at the time of diagnosis as well as incident complications over 2 years of follow-up will be compared with data from other studies. The data will be used to inform management strategies for patients diagnosed with diabetes in Zimbabwe.

## Introduction

Diabetes mellitus (diabetes) is a metabolic disorder that is characterized by hyperglycemia, which develops as a result of defective insulin secretion, insulin action, or both [1]. The burden of diabetes is increasing worldwide. In 2017, 15 million people were estimated to have diabetes in Africa, and this is projected to increase to 40.7 million by 2045 [2]. For sub-Saharan Africa (SSA) in 2017, there were an estimated 14.2 million people with diabetes, and this number is expected to rise to 43.2 million by 2040 [3]. As in other countries in SSA, the prevalence of diabetes in Zimbabwe is reported to have increased from 0.4% in the 1980s to 5.7% in 2014 [4]. Although the burden of diabetes is increasing worldwide, there is a dearth of information on the types of diabetes in SSA in general and in Zimbabwe, in particular, where classification is based mainly on age at presentation. In addition, there are no studies on the incidence of diabetes complications in Zimbabwe.

Classification of diabetes is evolving as more information about the disease is reported. Apart from the age at onset of diabetes, additional tests, such as C-peptide measurement, β-cell autoantibody testing, and genetic testing, may aid in the classification of diabetes and ultimately improve the care of patients through selection of appropriate therapy. The World Health Organization (WHO) and American Diabetes Association classify diabetes mellitus as type 1 (T1D), type 2 (T2D), hyperglycemia in pregnancy, unclassified diabetes, hybrid forms of diabetes, and other types of diabetes [1,5-8]. C-peptide is a useful biochemical measure for assessing pancreatic β-cell function, and it may be used to both classify the type of diabetes and to predict insulin requirement [9,10]. In a Scandinavian study, serum C-peptide >1.51 nmol/L was indicative of insulin resistance [11]. In a few studies reported from countries in SSA, including Ethiopia, Uganda, Sudan, and Côte d’Ivoire, patients with T2D were found to have high levels of circulating C-peptide. Measurement of C-peptide in these studies improved both the classification and treatment of patients [12]. Another useful investigation assisting in the classification of diabetes is β-cell autoantibody testing. The presence of 1 or more β-cell autoantibodies (glutamic acid decarboxylase, zinc transporter 8, insulinoma antigen, and islet cell antibody [ICA]) is suggestive of T1D [5,7]. Measurement of C-peptide and β-cell autoantibodies is expensive and not done routinely in Zimbabwe. Classification of diabetes in Zimbabwe is based on clinical criteria, including mode of presentation, age at diagnosis, and phenotype. Some adult patients may present with features of T1D, and similarly, young patients may present with clinical features of T2D. Misclassification of diabetes may result in the selection of inappropriate therapy. Therefore, this study was undertaken to assess the spectrum of diabetes mellitus and its complications at the time of diagnosis and to investigate the incidence and risk factors associated with the development of complications in a Zimbabwean population in Bulawayo. Establishing the spectrum of diabetes will hopefully assist in formulating policies on diabetes care in Zimbabwe.

The acute and chronic complications of diabetes are well described. Diabetic ketoacidosis (DKA) may be the mode of presentation of undiagnosed diabetes mellitus, and this acute metabolic complication is associated with a high mortality. Worldwide mortality due to DKA is high in T1D (20%), and in older people, it is responsible for deaths in 5%. In SSA, mortality due to DKA is estimated to be between 10% and 30% [8]. Furthermore, ketosis-prone type 2 diabetes (KPD) is reported in several African populations and may lead to confusion regarding the etiologic type of diabetes. KPD is characterized by the onset of diabetes with ketoacidosis, male predominance, low prevalence of β-cell antibodies, and high rates of either remission or insulin independence after initial stabilization [13]. Data on acute diabetes complications, in particular DKA, at the time of diagnosis in patients in Zimbabwe are not available.

Prolonged hyperglycemia leads to the development of chronic micro- (retinopathy, neuropathy, and nephropathy) and macro- (atherosclerosis) vascular complications of diabetes [14,15]. The prevalence of cardiovascular complications was reported to be 17.5%, and the presence of 1 complication was 24% in patients with newly diagnosed diabetes in a Swedish study using data from the Swedish National Diabetes register [16]. In the Verona study, approximately half of the participants had some chronic complications of diabetes at the time of diagnosis, 11.2% had cardiovascular disease complications, 21.2% had neuropathy, 4.9% had retinopathy, 8.8% had chronic kidney disease based on estimated glomerular filtration rate, and 13.2% had albuminuria [17]. In a systematic review of the prevalence of microvascular and macrovascular complications in patients with newly diagnosed T2D in low- to middle-income countries, Aikaeli et al [18] reported that the prevalence of ischemic heart disease was 10%, peripheral artery disease 6%, stroke 2%, diabetic foot 1%, neuropathy 16%, and microalbuminuria 24%. This study also highlighted the paucity of data from the Africa region, and of the 33 reports included for analysis, only 4 were from Africa (1 each from Nigeria and Mauritius and 2 from Egypt). A press release from the International Diabetes Federation 2023 reported that two-thirds of newly diagnosed diabetes patients present with a diabetes-related chronic complication [15,18].

Patients with newly diagnosed diabetes have been noted to present with diabetic foot or arm infection, which may result in amputation of the limb. The global prevalence of diabetic foot is 6.4%. In SSA, it ranges between 4% and 19% [19]. In a cross-sectional Zimbabwean clinic study on 69 patients with known diabetes, the prevalence of diabetic foot disease was 10% [20].

Owing to the lack of reported data on the mode of presentation, etiologic types of diabetes, and prevalence of diabetes complications at the time of diagnosis, this study was developed to fill knowledge gaps in Zimbabwe. In addition, incident complications over a 2-year follow-up period in patients with type 2 diabetes and no complications at diagnosis would provide further insight into the outcome of treatment of patients in Zimbabwe.

## Methods

This was a cross-sectional and prospective observational study undertaken on all adult and adolescent patients newly diagnosed with diabetes who presented to the diabetes clinical service at referral hospitals and 2 city clinics in Bulawayo, Zimbabwe.

### Study Design

This is a cross-sectional (baseline study, phase 1) and prospective (2-y follow-up study, phase 2) design.

The primary objective is to characterize the etiological types of diabetes in adult and adolescent patients with newly diagnosed diabetes in Zimbabwe.

The secondary objectives are to determine the prevalence of diabetes complications among adult and adolescent patients with newly diagnosed diabetes and the incidence and risk factors for the development of diabetic complications after a 2-year follow-up in patients with type 2 diabetes, who are complication free at baseline.

### Study Sites

The study was undertaken at the Diabetes Clinical Service unit at Mpilo Central Hospital, United Bulawayo Hospitals (UBH), and 2 health clinics in Bulawayo (Luveve and Mpopoma). Mpilo Central Hospital and UBH are tertiary referral hospitals for Bulawayo Metropolitan, Matabeleland North, Matabeleland South, Masvingo, and Midlands provinces. Luveve and Mpopoma clinics are in densely populated residential regions of the Bulawayo Metropolitan Province.

### Inclusion Criteria

The inclusion criteria were as follows: (1) patients aged ≥12 years presenting with newly diagnosed diabetes mellitus (WHO criteria) and (2) those who provided written consent or assent if aged <18 years.

### Exclusion Criteria

The exclusion criteria were as follows: (1) patients aged <12 years and (2) those who declined to participate in the study.

### Recruitment Strategy

The study was conducted at Mpilo Hospital, UBH, and 2 City Health Clinics. Messages regarding the study were disseminated through the hospital’s and the city health’s public relations offices to the public. The physicians were informed about the study through the Zimbabwe Medical Doctors Association and its affiliated members.

### Ethical Considerations

Informed consent and assent were obtained by the research team from the participant or legal guardian. Ethical clearance was granted by the Medical Research Council of Zimbabwe (MRCZ/A/2735) and the Biomedical Research Ethics Committee University of KwaZulu-Natal (BREC 00001442/2020). No compensation was given to the participants for enrolling into the study.

### Sample Size

In view of the limited number of studies on the prevalence of newly diagnosed diabetes in the region, a proportion of 0.3 (volume proxy figure) was used to calculate the sample size. Owing to time constraints, a pilot study could not be done. However, using the volume proxy proportion that was obtained in assessing the feasibility of the study, an analysis of patients’ registries seeking services at the health institutions where data were collected revealed that, on average, 3 adolescent and adult patients were newly diagnosed with diabetes every 10 days, translating to a prevalence rate of 30% per month. On the basis of the proportion (0.3) of patients with newly diagnosed diabetes mellitus seeking care at the 2 referral hospitals and 2 local clinics per month in Bulawayo, a margin of error of 5% and a Z score of 1.96 using the formula n=z^2^(pxq)/d^2^, the sample size for cross-sectional (phase 1) was estimated to be 323. The acceptability of the study could not be ascertained due to the paucity of research being conducted in our population.

According to the systematic analysis conducted by Aikaeli et al [18], the median prevalence of complications in patients with newly diagnosed diabetes ranged between 10% and 16%. Using a 16% prevalence rate of complications in patients with newly diagnosed diabetes and single group proportion design for binary outcomes, with 95% CI, 5% precision, 80% study power, and an attrition rate of 20% using the formula N=Z^2^xp(1−p)/^Δ2^=1.96^2^×0.16×(1−0.16)/0.05^2^=207 was the required sample size for the prospective study. After adjusting for a 20% rate for attrition or loss to follow-up, N*=N×1.25=259 was the calculated sample size. Assessment of the incidence of complications according to the type of treatment received was not assessed in this study. As this is a mixed utility design, the highest sample size of 323 was used to conduct the study.

### Statistical Analysis

SPSS 23 (IBM) was used to perform statistical analysis of the data. Descriptive statistical methods were used to analyze categorical variables such as types of diabetes, ankle-brachial index (ABI), carotid intima thickness, BMI, and autoantibodies. Continuous variables (eg, age, blood pressure, hemoglobin, cholesterol levels, random blood sugar, estimated glomerular filtration rate, glycated hemoglobin, and C-peptide levels) were documented as means and SDs. A chi-square analysis will be used to compare variables between males and females. The Cox regression analysis will be conducted to determine the time to the development of complications. Multiple regression analysis was used to identify risk factors for developing chronic complications of diabetes, and sensitivity analysis was used for the identification of the effect of confounders. Multiple imputation for missing values will be conducted to address the issue of missing values. Sensitivity analysis will be done during data analysis.

### Study Outline and Data Collection

#### Overview

**Figure 1.**
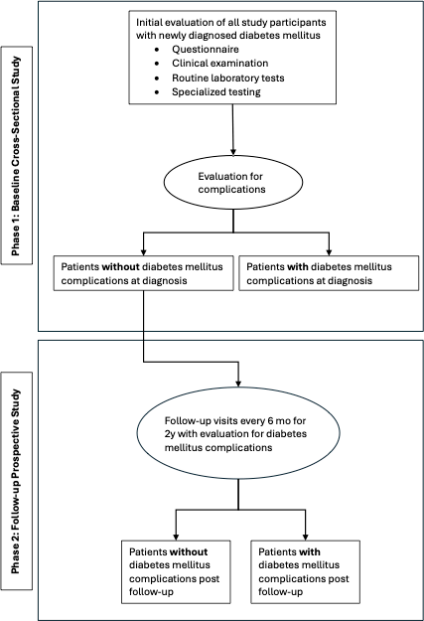
Study outline.

The study was undertaken in 2 phases. Adolescent and adult patients presenting to health care facilities with diabetes for the first time had their demographic data on age, sex, level of education, employment status, presenting symptoms, and medical history obtained using a questionnaire (Multimedia Appendix 1). Additionally, a physical examination and special tests such as ABI measurements, 10 g monofilament and 12 Hz tuning fork testing for neuropathy, and 12-lead electrocardiography (ECG) were performed by an experienced research team member. Carotid intima thickness measurements and retinal photography were done by a trained ultrasound sonographer and a specialist ophthalmologist, respectively. Patients without complications at diagnosis were enrolled into phase 2, and they were monitored for 2 years with clinical evaluations at every 6-month interval as shown in Figure 1. A summary of assessments of patients is presented in Textbox 1.

Textbox 1. **Summary assessment of participants with newly diagnosed diabetes.**

Questionnaire information

-  Education
-  Employment status
-  Alcohol use
-  Smoking
-  Presenting complaints
-  Medical history
-  Family history

Clinical examination

-  Blood pressure measurement
-  Height
-  Weight
-  Waist circumference
-  Hip circumference
-  Waist-to-hip ratio
-  10 g monofilament
-  128 Hz tuning fork

Special tests

-  Ankle-brachial index
-  Body composition assessment
-  Carotid intima thickness measurement
-  12-lead electrocardiograph
-  Retinal photography

Laboratory tests

-  Full blood count
-  Urea and electrolytes
-  Random blood glucose test
-  Capillary blood glucose test
-  Point-of-care glycated hemoglobin (HbA_1c_)
-  Laboratory HbA_1c_
-  Liver function test
-  High-density lipoprotein cholesterol
-  Low-density lipoprotein cholesterol
-  Triglycerides
-  Total cholesterol
-  Spot urine analysis

Special laboratory tests

- C-peptide
- Anti-GAD-65 antibody
- Zn 8 transporter antibody
- Islet cell antibody
- Anti-IA2 antibodies

#### Diagnostic Criteria for Diabetes, DKA, and Hyperosmolar Hyperglycemic Syndrome

Using WHO and American Diabetes Association recommendations, the diagnosis of diabetes mellitus was based on fasting plasma glucose ≥7.0 mmol/L, random plasma glucose ≥11.1 mmol/L in patients with symptoms characteristic of diabetes, and 2-hour plasma glucose ≥11.1 mmol/L during a 75 g oral glucose tolerance test [5,7]. DKA diagnosis was based on pH <7.3, HCO_3_ <18 mmol/L, and ≥2 ketones in the urine [21]. The hyperosmolar hyperglycemic syndrome diagnosis was made on the following criteria: blood glucose >33.3 mmol/L, serum osmolality ≥320 mOsm/kg, volume depletion with pH >7.3, and HCO_3_ >15.0 mmol/L [22].

Anthropometric measurements were performed using WHO guidelines [23,24].

#### BMI Measurements

The height and weight were used to calculate the BMI (weight [kg] divided by height [m^2^]). According to the WHO classification of BMI, BMI <18.5 kg/m^2^ is underweight, BMI between 18.5 and 24.9 kg/m^2^ is normal weight, BMI between 25.0 and 29.9 kg/m^2^ is overweight, BMI between 30.0 and 34.9 kg/m^2^ is class 1 obesity, BMI between 35 and 39.9 kg/m^2^ is class 2 obesity, and BMI ≥40 kg/m^2^ is class 3 obesity [23].

#### Waist Circumference

Using WHO and International Diabetes Federation recommendations for waist circumference (WC) cut points, WC is “elevated” if ≥94 cm in men and ≥80 cm in women; WC is “substantially elevated” if there are ≥102 cm in men and ≥88 cm in women, increasing both the metabolic and cardiovascular risks [24].

#### Hip Circumference

Hip circumference was measured in centimeters, with the individual standing with their feet together. A nonstretching tape measure was placed around the prominent part of the gluteal region parallel to the floor [24].

#### Waist-to-Hip Ratio

Waist-to-hip ratio (WHR) was obtained by dividing the waist measurement by the hip measurement. WHR is elevated if ≥0.90 in men and ≥0.85 in women. If WHR >1, the risk of cardiovascular disease is high [24].

#### Blood Pressure

Blood pressure was measured with the patient seated and after resting for at least 15 minutes. The blood pressure was measured 3 times, and the average was recorded as the BP, with BP ≥140/90 mm Hg defined as hypertension [25].

#### Body Mass Composition

The patient was requested to stand barefoot on the MC TANITA 780MA biomedical impedance machine, with both hands on the grip handle for 5 minutes. The visceral fat, body fat, muscle, bone, and water content values of the patient were measured and compared with gender- and age-specific values using the inbuilt machine formulae [26].

### Assessment for Chronic Complications

#### Diabetic Retinopathy

Assessment for background, proliferative diabetic retinopathy, and maculopathy was undertaken using ophthalmoscopy and retinal photography. In this study, nonmydriatic retinal photography was performed with a portable retinal camera (Microclear) by an ophthalmologist in all patients at diagnosis. Retinopathy was assessed using the Scottish grading system [27].

The Scottish grading system is as follows:

- R0: no retinopathy
- R1: mild background retinopathy (microaneurysms, flame exudates, >4 blot hemorrhages in one or both hemifields, and cotton wool spots)
- R2: moderate background diabetic retinopathy and >4 blot hemorrhages in one hemifield
- R3: severe nonproliferative or preproliferative diabetic retinopathy in both hemifields, intraretinal microvascular anomalies, and venous beading
- R4: Proliferative retinopathy: neovascularization of the disc, neovascularization elsewhere, vitreous hemorrhage, retinal detachment
- M0: No macular findings
- M1: Hard exudates within 1‐ to 2-disc diameters of fovea
- M2: Blot hemorrhages or hard exudates

#### Diabetic Neuropathy

Peripheral neuropathy was evaluated using a 10 g Semmes-Weinstein monofilament examination and vibration test. The monofilament was applied perpendicular to the pulp of the hallux, third toe, and metatarsal phalangeal joints 1, 2, and 3, for 2 seconds per site until it buckles. Absence of pressure sensation in more than 8 sites is regarded as the presence of peripheral neuropathy [28]. A 128 Hz tuning fork was placed at the interphalangeal joint of the hallux. The patient was asked to report any vibration felt. The normal sensation is felt for approximately 60 seconds. The vibration sensation was recorded as absent if the patient failed to perceive any vibration [29].

### Specialized Clinical Tests

#### Carotid Intima-Media Thickness

Carotid intima-media thickness (CIMT) was measured to assess for atherosclerosis using the DC7 Mindray ultrasound machine. A clear water-based gel was applied to the skin in the neck area above the carotid vessels. Each carotid vessel was assessed separately. A 6.5-MHz convex transducer was placed on the common carotid area, and the CIMT was measured. The normal CIMT ranges from 0.59 to 0.95 mm in men and 0.52 to 0.93 mm in women. CIMT >1 mm is associated with significant atherosclerosis [30]. The measurement of CIMT was undertaken by a qualified sonographer who was trained in CIMT measurements. To improve interrater reliability, CIMT measurements at all sites were carried out by 1 CIMT measurement–trained sonographer.

#### Ankle-Brachial Index

The patient was allowed to rest and lie in the supine position for 10 minutes. A blood pressure cuff was placed above the malleolus on the lower limb and above the cubital fossa on the arm. BP was measured twice in both limbs. The higher value of the 2 readings was used to calculate the ABI. ABI between 0.90 and 1.30 is normal, whereas ABI <0.90 is indicative of peripheral artery disease and ABI >1.30 is suggestive of calcification of the vessels [31]. To minimize errors in ABI measurements, all personnel underwent training.

#### Electrocardiography

A resting 12-lead ECG was recorded using a Schiller Cardiovit 102 machine. The patient was put in a supine position on a couch and had limb leads placed symmetrically. Chest leads were placed in order from V1 to V6, in the following sequence: V1 placed in the fourth intercostal space on the right sternal border, V2 on the fourth intercostal space on the left sternal border, V3 placed mid-way between V2 and V4, V4 placed on the fifth intercostal space midclavicular line, V5 placed between V4 and V6, and V6 placed on the fifth intercostal space midclavicular line.

#### Laboratory Tests

Blood samples were collected from all patients enrolled in the study (phase 1) by a trained laboratory team. Transportation of all samples was undertaken by the laboratory team following standard operating procedures using guidelines provided by the manufacturer of the reagents that were used to analyze specific samples.

Random plasma glucose, glycated hemoglobin, serum urea and electrolytes, serum antibodies, liver function tests, and random serum C-peptide were collected during the first visit [11]. Serum lipids were collected after 12 hours of fasting. Blood for random plasma glucose was collected in a sodium fluoride tube, and for all the other tests, a plain tube was used. A spot urine sample was collected at the initial visit in a plain tube. Laboratory blood tests are summarized in **Table 1**.

**Table 1.** Summary: laboratory investigations and methods of newly diagnosed diabetes.

Variable	Sample tube	Assay method
Full blood count	EDTA^a^ tube	Beckman Coulter
Random plasma glucose	Sodium fluoride (gray top)	Hexokinase (Vitros 5.1 analyzer)
HbA_1c_^b^ (point-of-care)	—^c^	Nephelometric method (point-of-care SDA_1C_ machine)
HbA_1c_	EDTA tube (purple top)	Calorimetric (MISPA I2)
Total cholesterol	Plain tube	Calorimetric (Vitros 5.1 analyzer)
Triglyceride	Plain tube	Calorimetric (Vitros 5.1 analyzer)
Cholesterol (HDL^d^)	Plain tube	Calorimetric (Vitros 5.1 analyzer)
Cholesterol (LDL^e^)	Plain tube	LDL calculated using Friedewald equation
Antiglutamic acid decarboxylase (anti-GAD^f^ 65)	Plain tube	ELISA^g^ (Euroimmun)
Anti-insulin IA2^h^ antibody	Plain tube	ELISA (Euroimmun)
Antizinc transporter 8 antibody	Plain tube	ELISA (Euroimmun)
Anti-islet antibody	Plain tube	Immunofluorescence
Serum C-peptide	Plain tube	MALGUMI C-Peptide (CLIA)
LFT^i^	Plain tube	
Total protein		Calorimetric (Vitros 5.1 analyzer)
Albumin		Calorimetric (Vitros 5.1 analyzer)
AST^j^		Multipoint rate (Vitros 5.1 analyzer)
ALT^k^		Multipoint rate (Vitros 5.1 analyzer)
ALP^l^		Multipoint rate (Vitros 5.1 analyzer)
LDH^m^		Multipoint rate (Vitros 5.1 analyzer)
Total bilirubin		Micro Sensor TM (Vitros 5.1 analyzer)
HIV	EDTA tube (red top)	ELISA
CD4 count	EDTA	Flow cytometry (BD FACSCount)
Spot urine and microalbumin or UACR^n^	Plain tube	Spectrophotometry (Vitros 5.1)

a EDTA: ethylenediaminetetraacetic acid.

b HbA1c: glycated hemoglobin.

c Not available.

d HDL: high-density lipoprotein.

e LDL: low-density lipoprotein.

f GAD: glutamic acid decarboxylase.

g ELISA: enzyme-linked immunosorbent assay.

h IA2: insulinoma antigen 2.

i LFT: liver function test.

j AST: aspartate transaminase.

k ALT: alanine transaminase.

l ALP: alkaline phosphatase.

m LDH: lactate dehydrogenase.

n UACR: urine albumin-creatinine ratio.

### Prospective Study—Phase 2

The prospective (phase 2) component of the study includes all patients with newly diagnosed type 2 diabetes who were without chronic complications at initial presentation (phase 1).

Patients with any diabetes-related complication at presentation were excluded. Follow-up visits at 6, 12, 18, and 24 months after diagnosis aimed to assess for incident diabetes complications.

Similar to the baseline study, at every visit during follow-up, the methodology included questionnaire information, clinical examination, and laboratory investigations; additionally, assessment of medications and diabetes control was undertaken. The questionnaire used in phase 1 was used to assess the incidence of chronic diabetes complications. Retinal examination, body composition, carotid intimal thickness, and ECG were performed at 12 and 24 months. The follow-up of patients and outcomes during the clinic visits are summarized in Table 2. Diabetes complications that occurred between the scheduled visits were reported to the research team by the attending health care personnel. Adverse events related to prescribed medicines were addressed by the attending medical team.

**Table 2.** Summary of phase 2 visits and outcomes.

Parameter being assessed	Normal ranges	Baseline	6 mo	12 mo	18 mo	24 mo
Diabetes care	—^a^	—	Insulin or oral antidiabetic agent	Insulin or oral antidiabetic agent	Insulin or oral antidiabetic agent	Insulin or oral antidiabetic agent
BMI (kg/m^2^)	18.5‐24.9	✓^b^	✓	✓	✓	✓
SBP^c^ (mm Hg)	<140	✓	✓	✓	✓	✓
DBP^d^ (mm Hg)	<90	✓	✓	✓	✓	✓
ABI^e^	0.90‐1.30	✓	✓	✓	✓	✓
10 g monofilament	Normal sensation in 8 or >8 sites	✓	✓	✓	✓	✓
HbA_1c_^f^ (%/mmol/mol)	<5.7/42	✓	✓	✓	✓	✓
Total cholesterol (mmol/L)	0‐5.2	✓	✓	✓	✓	✓
LDL^g^ (mmol/L)	<2.0	✓	✓	✓	✓	✓
HDL^h^ (mmol/L)	1.03‐1.55	✓	✓	✓	✓	✓
Triglyceride (mmol/L)	0‐1.69	✓	✓	✓	✓	✓
Urea (mmol/L)	2.5‐7.9	✓	✓	✓	✓	✓
Creatinine (μmol/L)	55‐120	✓	✓	✓	✓	✓
eGFR^i^ (mL/min/1.73m^2^)	≥90	✓	✓	✓	✓	✓
Urinalysis	No abnormality	✓	✓	✓	✓	✓
CIMT^j^ (mm)	0.6‐0.7	✓		✓		✓
Retinal photograph	Normal	✓		✓		✓
ECG^k^	Normal	✓		✓		✓

a Not applicable.

b Checkmarks indicate which measurements are taken at each time point.

c SBP: systolic blood pressure.

d DBP: diastolic blood pressure.

e ABI: ankle-brachial index.

f HbA_1c_: glycated hemoglobin.

g LDL: low-density lipoprotein.

h HDL: high-density lipoprotein.

i eGFR: glomerular filtration rate.

j CIMT: carotid intima media thickness.

k ECG: electrocardiograph.

## Results

The study describes the characteristics of patients with newly diagnosed diabetes, including the mode of presentation, the prevalence of complications, and the assignment of a specific etiologic type, in patients presenting to 4 urban health facilities in Bulawayo, Zimbabwe. In addition, the prevalence of diabetes complications at the time of diagnosis and the incidence of diabetes complications over 2 years of postdiagnosis follow-up will be determined (Textbox 1). Data collection for the cross-sectional study (phase 1) commenced in October 2021 and ended in October 2024. Data analysis and manuscript writing for the phase 1 study are underway. Data collection for the prospective study (phase 2) is ongoing until October 2026.

## Discussion

### Principal Findings

Epidemiologic data indicate a rising prevalence of diabetes in Zimbabwe [4], as in other countries in SSA, and therefore, the health services would expect to encounter patients with symptomatic disease more frequently [3]. However, it is not known what the common mode of presentation is in Zimbabwe, nor are there data available on the prevalence of microvascular and macrovascular complications at the time of diagnosis. There are several factors that may influence the increasing prevalence of diabetes, including urbanization, obesity, diet and lifestyle changes, environmental toxins, alcohol, and other factors [2]. The influence of these factors in Zimbabwe is not known, and this study will provide some information in this regard, as the data on lifestyle, mode of presentation, and clinical phenotype will provide details on the association with obesity and lifestyle. In addition, this study incorporates patients from 12 years of age, and it is envisaged that a significant proportion of the younger patients will be diagnosed with type 1 diabetes. The prevalence of type 2 diabetes in young persons in Zimbabwe is not known, and this study will provide details on this diagnosis. Similarly, less common etiologic types, such as ketosis-prone diabetes, may be a form of the disease in Zimbabwe. This study uses diagnostic methods that will assist in assigning an etiology with a level of certainty much higher than has been the case in the past, where reliance was primarily on age at diagnosis and clinical phenotype. While C-peptide may be affected by acute, severe hyperglycemia, due to β-cell glucose toxicity, the combination of both C-peptide and measurement of β-cell autoantibodies will provide a level of diagnostic clarity that has not been possible in the past [5,7,10-12]. It is envisaged that younger patients may have type 1 diabetes, and the lean adults presenting with ketoacidosis may have KPD. As with most other studies, it is considered likely that the majority of the remaining subjects enrolled will have type 2 diabetes. The study will provide data on the body composition of subjects with different etiologic types of diabetes, and while obesity may be increasing in parallel with diabetes, it is not known whether or not this occurs in patients with newly diagnosed diabetes in Zimbabwe. Furthermore, the prevalence of common comorbidities, including hypertension and dyslipidemia, will be provided by the initial phase of this study.

It is possible that there is a high prevalence of undiagnosed diabetes mellitus in Zimbabwe and that only once symptoms develop will the diagnosis be established. This implies prolonged undiagnosed hyperglycemia, during which time complications may develop [14,17,18]. This study will provide data on the prevalence of complications, using investigations, such as retinal photography and carotid intima-media thickness, as well as more widely used clinical tools to improve the detection of these complications. Accurate diagnosis of diabetes complications is important because the implementation of medical management strategies to mitigate progression will alleviate some of the burden on the health care system. Furthermore, the prospective component of the study will provide more data on the incidence of complications in those with type 2 diabetes.

### Limitations

There are several limitations to this study. Patients presenting to the selected 4 health care facilities in Bulawayo may not reflect the broader population of Zimbabwe, particularly those residing entirely in rural areas. The study is only able to report on clinical, laboratory, and radiologic parameters in patients presenting to the health services with symptomatic disease and cannot determine factors associated with undiagnosed disease. The follow-up period in the prospective study is relatively short and therefore will not provide a true estimate of the long-term risk of complications. There may be an attrition of follow-up attendance in the prospective phase, but steps will be taken to minimize this, including direct contact with the patients in this phase of the study.

### Strengths

The strengths of this study include detailed characterization of the enrolled patients with sophisticated laboratory and radiologic techniques that have not been used in prior studies in Zimbabwe as well as the detailed follow-up evaluation of patients to determine not only incident complications but also adequacy of glycemic control and management of comorbidities.

The importance of this study to public health in Zimbabwe is envisaged as raising awareness of the common modes of presentation and etiologic types of diabetes and informing health authorities and health practitioners to assist in the more appropriate therapeutic measures to use, with the aim of lowering the development of chronic complications.

## Supplementary material

10.2196/74186 Multimedia Appendix 1 Questionnaire and clinical examination.

## Abbreviations

ABI: ankle-brachial index
CIMT: carotid intima-media thickness
DKA: diabetic ketoacidosis
ECG: electrocardiograph
HbA_1c_: glycated hemoglobin
KPD: ketosis-prone type 2 diabetes
SSA: sub-Sahara Africa
T1D: type 1 diabetes
T2D: type 2 diabetes
WC: waist circumference
WHO: World Health Organization
WHR: waist-to-hip ratio

## References

[1] Alberti KG, Zimmet PZ (1998). Definition, diagnosis and classification of diabetes mellitus and its complications. Part 1: diagnosis and classification of diabetes mellitus provisional report of a WHO consultation. Diabet Med.

[2] (2015). The Diabetes Atlas, 7th edn. https://www.diabetesatlas.org.

[3] (2017). The Diabetes Atlas, 8th edn. https://www.diabetesatlas.org.

[4] Mutowo M, Gowda U, Mangwiro JC, Lorgelly P, Owen A, Renzaho A (2015). Prevalence of diabetes in Zimbabwe: a systematic review with meta-analysis. Int J Public Health.

[5] American Diabetes Association (2019). 2. Classification and diagnosis of diabetes: standards of medical care in diabetes-2019. Diabetes Care.

[6] (1999). Definition, diagnosis and classification of diabetes mellitus and its complications: report of a WHO consultation. part 1, diagnosis and classification of diabetes mellitus. https://iris.who.int/handle/10665/66040.

[7] (2019). Classification of diabetes mellitus. https://iris.who.int/handle/10665/325182.

[8] Mbanya JCN, Motala AA, Sobngwi E, Assah FK, Enoru ST (2010). Diabetes in sub-Saharan Africa. Lancet.

[9] Jones AG, Hattersley AT (2013). The clinical utility of C-peptide measurement in the care of patients with diabetes. Diabet Med.

[10] Zhou Patricia D, Kouassi JB, Monteomo GF (2017). Basic C-peptidemia and diabetic patients classification. Sci J Clin Med.

[11] Berger B, Stenström G, Sundkvist G (2000). Random C-peptide in the classification of diabetes. Scand J Clin Lab Invest.

[12] Gill GV, Tekle A, Reja A (2011). Immunological and C-peptide studies of patients with diabetes in northern Ethiopia: existence of an unusual subgroup possibly related to malnutrition. Diabetologia.

[13] Umpierrez GE, Smiley D, Kitabchi AE (2006). Narrative review: ketosis-prone type 2 diabetes mellitus. Ann Intern Med.

[14] Sosale A, Prasanna Kumar KM, Sadikot SM (2014). Chronic complications in newly diagnosed patients with type 2 diabetes mellitus in India. Indian J Endocrinol Metab.

[15] (2021). The Diabetes Atlas, 10th. https://www.diabetesatlas.org.

[16] Höskuldsdóttir G, Franzén S, Eeg-Olofsson K, Eliasson B (2022). Risk trajectories of complications in over one thousand newly diagnosed individuals with type 2 diabetes. Sci Rep.

[17] Bonora E, Trombetta M, Dauriz M (2020). Chronic complications in patients with newly diagnosed type 2 diabetes: prevalence and related metabolic and clinical features: the Verona newly diagnosed type 2 diabetes study (VNDS) 9. BMJ Open Diabetes Res Care.

[18] Aikaeli F, Njim T, Gissing S (2022). Prevalence of microvascular and macrovascular complications of diabetes in newly diagnosed type 2 diabetes in low-and-middle-income countries: a systematic review and meta-analysis. PLOS Glob Public Health.

[19] Abbas ZG, Archibald LK (2005). Epidemiology of the diabetic foot in Africa. Med Sci Monit.

[20] Nyika P, Chimusoro A, Tshimanga M, Gombe N, Takundwa L, Bangure D (2015). Risk factors for diabetic complications among diabetic patients, Chirumanzu District, Zimbabwe, 2011. Austin J Public Health Epidemiol.

[21] Umpierrez GE, Davis GM, ElSayed NA (2024). Hyperglycemic crises in adults with diabetes: a consensus report. Diabetes Care.

[22] Fadini GP, de Kreutzenberg SV, Rigato M (2011). Characteristics and outcomes of the hyperglycemic hyperosmolar non-ketotic syndrome in a cohort of 51 consecutive cases at a single center. Diabetes Res Clin Pract.

[23] (2025). Body mass index (BMI). World Health Organization.

[24] (2011). Waist circumference and waist-hip ratio: report of a WHO expert consultation, Geneva, 8-11 December 2008. https://iris.who.int/handle/10665/44583.

[25] Whelton PK, Carey RM, Aronow WS (2018). 2017 ACC/AHA/AAPA/ABC/ACPM/AGS/APhA/ASH/ASPC/NMA/PCNA guideline for the prevention, detection, evaluation, and management of high blood pressure in adults: executive summary: a report of the American College of Cardiology/American Heart Association Task Force on Clinical Practice Guidelines. Hypertension.

[26] Tanita.

[27] Zachariah S, Wykes W, Yorston D (2015). Grading diabetic retinopathy (DR) using the Scottish grading protocol. Community Eye Health.

[28] Boulton AJM, Armstrong DG, Albert SF (2008). Comprehensive foot examination and risk assessment: a report of the task force of the foot care interest group of the American Diabetes Association, with endorsement by the American Association of Clinical Endocrinologists. Diabetes Care.

[29] Gilman S (2002). Joint position sense and vibration sense: anatomical organisation and assessment. J Neurol Neurosurg Psychiatry.

[30] Chen LY, Leening MJG, Norby FL (2016). Carotid intima-media thickness and arterial stiffness and the risk of atrial fibrillation: the atherosclerosis risk in communities (ARIC) study, multi-ethnic study of atherosclerosis (MESA), and the Rotterdam study. J Am Heart Assoc.

[31] Aboyans V, Criqui MH, Abraham P (2012). Measurement and interpretation of the ankle-brachial index: a scientific statement from the American Heart Association. Circulation.

