# Supplementary material for: Etiologic Types and Complications of Diabetes Mellitus in Newly Diagnosed Patients at Health Institutions in Bulawayo, Zimbabwe: Protocol for a Cross-Sectional and Prospective Observational Study
Source: JMIR Res Protoc. 2026 Jan 6;15:e74186. doi: 10.2196/74186 (PMC12774401; doi:10.2196/74186)
Supplement: Multimedia Appendix 1 [file resprot-v15-e74186-s001.pdf]

Appendix 1: Data collection tool for newly diagnosed diabetes patients.

Hospital number	Date of enrolment into the study
Date of birth	Study Number
Age	Gender
Date of diagnosis (diabetes)	Marital status
Employment status/self-employed/ employed/unemployed	Level of education/primary/secondary/tertiary

History

Polyuria Yes ☐ No ☐

Polydipsia Yes ☐ No ☐

Irritability Yes ☐ No ☐

Fatigue Yes ☐ No ☐

Vulval itchiness Yes ☐ No ☐

Nausea/vomiting Yes ☐ No ☐

Abdominal pain Yes ☐ No ☐

Weight loss or gain Yes ☐ No ☐

Numbness Yes ☐ No ☐

Loss of visual acuity Yes ☐ No ☐

Skin infections Yes ☐ No ☐

Erectile dysfunction/libido Yes ☐ No ☐

Excessive hunger Yes ☐ No ☐

Past medical history

Hypertension Yes ☐ No ☐

Connective tissue Yes ☐ No ☐

Chronic pancreatitis Yes ☐ No ☐

Gestational diabetes Yes ☐ No ☐

Polycystic ovarian syndrome Yes ☐ No ☐

Other disease

Medications used in the past

Use steroids Yes ☐ No ☐

Thiazide Yes ☐ ☐

Antipsychotics Yes ☐ No ☐

Cytotoxic Yes ☐ ☐

Social habits

Smoking Yes ☐ Pack Year No ☐

Alcohol Yes ☐ units per week No ☐

Clinical examination and vital signs

Anthropometry

☐ Height in cm

☐ Weight in kg

☐

BMI Kg/m^2

Waist circumference in (cm)

Hip circumference in (cm)

Waist hip ratio (WHR)

General examination

Anaemia Yes No

Jaundice Yes No

Xanthoma Yes No

Acanthosis nigricans Yes No

Skin infections Yes No

Hydration status Normal Abnormal

Sweaty palms Yes No

Examination of the oral cavity

Vital signs Respiratory rate heart rate Systolic BP

Diastolic BP

Cardiovascular System

Orthostatic hypotension Yes No

Respiratory system

Abdomen

Neurology

Fundal examination

Cranial Nerves

Motor

Sensory

Urine dipstick
